# No change in the severity of eating disorders in Japanese children during the COVID‐19 pandemic

**DOI:** 10.1002/pcn5.237

**Published:** 2024-08-15

**Authors:** Masahide Usami, Yoshinori Sasaki, Kotoe Itagaki, Yuta Yoshimura, Kumi Inazaki, Yuki Hakoshima, Yuki Mizumoto

**Affiliations:** ^1^ Department of Child and Adolescent Psychiatry, Kohnodai Hospital National Center for Global Health and Medicine Ichikawa Chiba Japan; ^2^ Department of Psychiatry and Behavioral Science Tokyo Medical and Dental University Tokyo Japan; ^3^ Department of Clinical Psychology, Kohnodai Hospital National Center for Global Health and Medicine Ichikawa Chiba Japan; ^4^ Department of Psychiatry Fukuoka University Fukuoka Fukuoka Fukuoka Japan

In Japan, children and adults were living with social restrictions such as curfews, lockdowns, social distancing, travel restrictions, and several times vaccination during the COVID‐19 pandemic. Staying home and being less active outdoors can lead to less physical activity, social withdrawal from one's usual surroundings, reduced companionship, more screen time, irregular sleep patterns, and worse eating habits.^1^ The secondary effects of social isolation and loneliness in all ages include various reactive psychiatric symptoms.^2^ As the pandemic has now persisted for more than 2 years, some of these symptoms may have led to changes in eating behaviors. A previous study reported an increase in both dietary and binge eating behaviors in a community sample during the COVID‐19 pandemic.^3^ In addition, patients with anorexia nervosa have reported increased restriction and anxiety about eating during the COVID‐19 pandemic.^4^ Furthermore, eating disorders (EDs) are reported to be rapidly increasing in child and adolescent psychiatric practices in Japan.^5^, ^6^ However, it was unknown if the physical condition of children with EDs had deteriorated compared to pre‐pandemic.

To understand the situation during the pandemic in Japan, we compiled clinical information about elementary and junior high school students with EDs from the Child and Adolescent Mental Health Registry. This Registry is approved by the Ethics Committee of National Center for Global Health and Medicine (NCGM) and includes all clinical information about children who visited the Department of Child and Adolescent Psychiatry, Kohnodai Hospital, NCGM, since January 2016. Withdrawal of consent to this registry was obtained by opting out, and the database was anonymized. The dataset included a total of 142 children diagnosed with an ED, 66 of whom visited the clinic before February 2020 (62 months) and 77 of whom visited the clinic during the period of March 2020 to December 2021 (22 months). Patients were divided into two groups: before and after the COVID‐19 pandemic period. This study defined the pandemic period as starting in March 2020 based on the decision made by the government Headquarters for COVID‐19 response on February 25, 2020.

Table 1 reports the number of children with ED, average age, gender, school, body mass index (BMI), inpatient/outpatient status, and referral rate from other medical institutions for the two groups. Before February 2020 (pre‐pandemic period), 65 children (57 girls/eight boys) with an ED visited the NCGM. Their age and BMI were 12.3 ± 1.9 years and 14.5 ± 2.1, respectively. The 20 children in this group were inpatients (30.7%). After March 2020 (pandemic period), 77 children (76 girls/one boy) with an ED visited the NCGM. Their age and BMI were 13.0 ± 1.6 years and 14.7 ± 2.3, respectively. The 18 children of this group were inpatients (23.3%). The pre‐pandemic and pandemic groups were then statistically compared by two‐factor analysis of variance for each school group and gender. Compared to the pre‐pandemic period, the number of children with ED per month in the pandemic period showed a 3.2‐fold increase from 1.1 to 3.5. No significant differences were found in the BMIs of elementary school students with EDs, because the BMIs of pre‐adolescent children are lower than in adolescence and adulthood.^5^ The lower BMIs of junior high school girls requiring hospitalization did not change. However, there were no significant differences in BMIs or average age between the two groups, suggesting that the COVID‐19 pandemic did not result in younger or more severe physical problems in children with EDs.

**Table 1:** Age and BMI of eating disorders in the pre‐pandemic group and pandemic groups

	Age			*p* value		BMI																														
						Elementary school students								Total			*p* value		Junior high school students								Total			*p* value		Total			*p* value	
						Girls			*p* value		Boys								Girls			*p* value		Boys												
	M	SD	*n*			M	SD	*n*			M	SD	*n*	M	SD	*n*			M	SD	*n*			M	SD	*n*	M	SD	*n*			M	SD	*n*		
Pre‐Pandemic	12	1.9	65		NS	13.1	1.3	19		NS	14.6	0.5	3	13.3	1.3	22		NS	15.0	2.2	38		NS	16.2	1.4	5	15.1	2.1	43		NS	14.5	2.1	65		NS
Outpatients	13	1.9	45	[**]		12.7	1.3	12	NS		14.9	0	1	12.9	1.4	13	NS		15.3	2.4	27	[*]		16.2	1.4	5	15.4	2.3	32	[**]		14.7	2.4	45	[**]	
Inpatients	12.0	1.8	20			13.8	0.8	7			14.5	0.6	2	13.9	0.8	9			14.2	1.2	11			0	0	0	14.2	1.2	11			14.1	1.1	20		
Pandemic	13.0	1.6	77			13.3	2.3	14			14.7	0	1	13.4	2.3	15			15.0	2.2	62			0	0	0	15.0	2.2	62			14.7	2.3	77		
Outpatients	13	1.5	59	[**]		13.9	3.2	6	NS		14.7	0	1	14.0	3.0	7.0	NS		15.3	2.2	52	[*]		0	0	0	15.3	2.2	52	[**]		15.1	2.4	59	[**]	
Inpatients	12	1.6	18			12.8	0.9	8			0	0	0	12.8	0.9	8			13.7	1.2	10			0	0	0	13.7	1.2	10			13.3	1.2	18		
Total	13	1.8	142			13.2	1.8	33			14.6	5	4	13.3	1.8	37								16.2	1.4	5	15	2.1	10			14.6	2.2	142		

M, mean; *n*, number; NS, not significant; SD, standard deviation.

* *p* < 0.05,

** *p* < 0.001

This analysis did not address any psychosocial factors or comorbid disorders in these patients. Further investigation in multiple institutions and countries is thus needed to address various other factors, such as triggers of ED and social circumstances. Social changes for children in Japan were the closure of schools and that parents worked from home during the pandemic.

These findings suggested that although the number of children with EDs is increasing, their conditions did not become more severe during the pandemic. Some children with EDs are admitted to the hospital. As there are few specialized wards for child and adolescent psychiatry in Japan, an increasing number of children with EDs will make it more difficult to treat children with other mental disorders, such as neurodevelopmental disorders, severe self‐injurious or suicide attempts. During the pandemic, the number of patients with ED in pediatric departments increased and their severity was worse in Japan and other country.^7^, ^8^ The limitation of this study was the use of data from one institution, and that this finding must be considered in the context of other institutions' pediatric or emergency departments treating children with ED in life‐threatening situations.

## AUTHOR CONTRIBUTIONS

Masahide Usami designed this study. Masahide Usami, Yuta Yoshimura, Kumi Inazaki, Yuki Hakoshima, and Yuki Mizumoto collected the data. Masahide Usami and Yoshinori Sasaki analyzed the data.

## CONFLICT OF INTEREST STATEMENT

The authors declare no conflict of interest.

## ETHICS APPROVAL STATEMENT

This study was approved by National Center for Global Health and Medicine (NCGM‐S‐004487‐03).

## PATIENT CONSENT STATEMENT

Written or verbal informed consent was not obtained because the study was conducted according to the Ethical Guidelines for Medical and Health Research Involving Human Subjects of Japan. The guidelines state that it is not always necessary to obtain informed consent from study participants.

## CLINICAL TRIAL REGISTRATION

None

## Data Availability

None
